# Disruption of Cationic/Anionic Viscoelastic Surfactant Micellar Networks by Hydrocarbon as a Basis of Enhanced Fracturing Fluids Clean-Up

**DOI:** 10.3390/nano10122353

**Published:** 2020-11-27

**Authors:** Andrey V. Shibaev, Anna L. Aleshina, Natalya A. Arkharova, Anton S. Orekhov, Alexander I. Kuklin, Olga E. Philippova

**Affiliations:** 1 Physics Department, Lomonosov Moscow State University, 119991 Moscow, Russia; aleshina@polly.phys.msu.ru (A.L.A.); phil@polly.phys.msu.ru (O.E.P.); 2 A.V. Shubnikov Institute of Crystallography, 119333 Moscow, Russia; natalya.arkharova@yandex.ru; 3 National Research Centre “Kurchatov Institute”, 123182 Moscow, Russia; orekhov.anton@gmail.com; 4 Moscow Institute of Physics and Technology, 141701 Dolgoprudny, Russia; kuklin@nf.jinr.ru; 5 Frank Laboratory of Neutron Physics, Joint Institute for Nuclear Research, 141980 Dubna, Russia

**Keywords:** viscoelastic surfactant solutions, wormlike surfactant micelles, microemulsion, enhanced oil recovery, hydraulic fracturing

## Abstract

Studies of the effects produced by the solubilization of hydrophobic substances by micellar aggregates in water medium are quite important for applications of viscoelastic surfactant solutions for enhanced oil recovery (EOR), especially in hydraulic fracturing technology. The present paper aims at the investigation of the structural transformations produced by the absorption of an aliphatic hydrocarbon (n-decane) by mixed wormlike micelles of cationic (n-octyltrimethylammonium bromide, C8TAB) and anionic (potassium oleate) surfactants enriched by C8TAB. As a result of contact with a small amount (0.5 wt%) of oil, a highly viscoelastic fluid is transformed to a water-like liquid. By small-angle neutron scattering (SANS) combined with cryo-TEM, it was shown that this is due to the transition of long wormlike micelles with elliptical cross-sections to ellipsoidal microemulsion droplets. The non-spherical shape was attributed to partial segregation of longer- and shorter-tail surfactant molecules inside the surfactant monolayer, providing an optimum curvature for both of them. As a result, the long-chain surfactant could preferably be located in the flatter part of the aggregates and the short-chain surfactant—at the ellipsoid edges with higher curvature. It is proven that the transition proceeds via a co-existence of microemulsion droplets and wormlike micelles, and upon the increase of hydrocarbon content, the size and volume fraction of ellipsoidal microemulsion droplets increase. The internal structure of the droplets was revealed by contrast variation SANS, and it was shown that, despite the excess of the cationic surfactant, the radius of surfactant shell is controlled by the anionic surfactant with longer tail. These findings open a way for optimizing the performance of viscoelastic surfactant fluids by regulating both the mechanical properties of the fluids and their clean-up from the fracture induced by contact with hydrocarbons.

## 1. Introduction

Many ionic surfactants are able to form very long semi-flexible cylindrical aggregates, called wormlike micelles (WLMs) [1,2,3]. Usually, it occurs upon addition of low-molecular weight salt [4,5,6,7,8,9] or an oppositely charged surfactant [10,11,12,13,14,15,16,17,18,19,20,21], which screen the electrostatic repulsion between charged surfactant head groups. The screening favors closer packing of surfactant ions and decreases the energy of cylindrical fragments of micelles as compared to their semi-spherical end-caps, which results in the growth of micelles in length.

Wormlike surfactant micelles have attracted considerable attention over recent years, which is due to their remarkable rheological properties [1,2,3,12]. It was shown that micellar chains can entangle and form a network, imparting high viscoelasticity to water solutions. Since the WLMs are formed by weak non-covalent interactions, their viscoelastic properties are very sensitive to various external triggers [1]. Of particular interest is the responsiveness of WLMs to added non-polar substances, for instance, hydrocarbons. It was demonstrated that hydrocarbons absorbed in the micellar core induce the transition of WLMs into microemulsion droplets, which is accompanied by a sharp drop of viscosity by several orders of magnitude and a complete disappearance of viscoelastic properties [22,23,24,25,26]. This behavior inherent to WLMs is of primary importance for oil industry [27,28,29,30,31,32,33,34,35], since it permits to clean up the formations from the residuals of surfactants that are left after the treatment of the well (drilling, fracturing, etc.). As a result of high responsiveness of viscoelastic surfactant fluids to hydrocarbons, they usually do not require addition of internal breakers [36] (contrary to polymer-based fluids), since their viscosity is sufficiently decreased upon contact with formation hydrocarbons. This minimizes contamination of the proppant pack by residues left after incomplete breaking of the fluid.

The mechanism of single-surfactant ionic WLMs transformation upon absorption of non-polar substances was studied both experimentally [23,24,31,33,37,38] and theoretically [24]. On an example of aliphatic hydrocarbons, it was shown that for linear WLMs the solubilization occurs preferentially in the energetically unfavorable end-caps of the micelles stabilizing them. This induces the shortening of the micelles and finally the formation of microemulsion droplets [24].

In most of the studies, the resulting microemulsion droplets are always of spherical shape [23,24,39] because of the domination of the surface tension over all other forces affecting the shape. However, recently for the first time the formation of non-spherical (ellipsoidal) microemulsion droplets upon solubilization of oil by WLMs was demonstrated [25]. It occurred when a mixture of cationic (n-octyltrimethylammonium bromide (C8TAB)) and anionic (potassium oleate) surfactants strongly differing in the hydrophobic tail lengths was used under conditions, when the longer-chain anionic surfactant was in excess. It was suggested that in that system the microemulsion droplets of ellipsoidal shape were formed, because they provided an optimum curvature of the surfactant monolayer both for long-chain surfactants (in the flatter part of the droplet) and for short-chain surfactants (at the edges with higher curvature). The formation of non-spherical microemulsion particles may affect the hydraulic fracturing process. For instance, non-spherical particles can pack more densely and behave differently, e.g., under the shear flow [40], which occurs during the outflow of the broken fluid upon the fracture clean-up.

Up to now, in most of the works, the effect of oils on wormlike micelles formed by a single ionic surfactant (anionic or cationic) have been studied. At the same time, mixed ionic WLMs are now starting to attract significant attention as fracturing fluids [41,42,43], especially for use in low-salinity and high-temperature reservoirs [44], because of their low critical micelle concentrations leading to lower consumption of surfactants, ultralow interfacial tension, and high oil solubilization. However, the effect of oils on such mixed micelles has been little studied.

In this article, we investigate the transformation of WLMs to microemulsion droplets for a mixture of cationic (C8TAB) and anionic (potassium oleate) surfactants strongly differing in the hydrophobic tail lengths under conditions, when the shorter-chain cationic surfactant is in excess. We show that such WLMs demonstrate an interesting rheological behavior, combining high viscoelasticity with a very short relaxation time. We demonstrate that this system forms spontaneously ellipsoidal microemulsion upon addition of hydrocarbon. In addition, by using small-angle neutron scattering (SANS) with contrast variation, for the first time we reveal the internal structure of the ellipsoidal droplets.

## 2. Materials and Methods

### 2.1. Materials

Potassium oleate (>98%) from TCI (Tokyo, Japan), n-octyltrimethylammonium bromide C8TAB (>98%) from ABCR (Karlsruhe, Germany), n-decane (>99%) from Sigma Aldrich (St. Louis, MO, USA) and potassium hydroxide (>98%) from Acros (Geel, Belgium) were used as received. Solutions were prepared using distilled deionized water purified by a Milli-Q system (Millipore, Burlington, MA, USA). D_2_O from AstraChem (Saint-Petersburg, Russia, 99.9% isotopic purity) and d-decane (Sigma Aldrich, 99% isotopic purity) were used for the preparation of the samples for SANS studies.

### 2.2. Samples Preparation

First, stock solutions of C8TAB and potassium oleate in water were prepared. The pH of the solutions was adjusted to 11.0 ± 0.1 by adding potassium hydroxide solution. Then the stock solutions were mixed in appropriate quantities with 10^−3^ M KOH (pH 11.0) to get the required final concentration of the solution and left to equilibrate at room temperature for several days. To investigate the effect of hydrocarbon, a required quantity of n-decane was added to the surfactant solution, and the resulting system was mixed by a magnetic stirrer overnight and left to equilibrate for few days.

### 2.3. Rheometry

The rheological measurements were carried out on a stress-controlled rotational rheometer Physica MCR 301 (Anton Paar, Gra, Austria) as described elsewhere [45]. For viscous samples with zero-shear viscosity η_0_ > 0.1 Pa·s a cone-plate geometry (diameter 40 mm, cone angle 2°) was used, whereas for samples with lower viscosity the measurements were performed in double gap coaxial cylinders (mean diameter 26.4 mm, height 40 mm, gap 0.42 mm). In both cases, a special cover was used to prevent solvent evaporation during the experiments. Temperature was set at 20.00 ± 0.05 °C by Peltier elements.

In steady shear (static) experiments, the shear stress was varied in the range of 0.005–10 Pa. The zero-shear viscosity was determined as the value of viscosity on the plateau at low stress. Oscillatory shear (dynamic) measurements were taken over the angular frequency ω range of 0.4–300 rad/s in the linear viscoelastic regime so that the storage and loss moduli were independent of deformation amplitude.

### 2.4. Small-Angle Neutron Scattering (SANS)

SANS experiments were performed at the IBR-2 pulsed reactor, Frank Laboratory of Neutron Physics, Joint Institute for Nuclear Research (Dubna, Russia) on the YuMO spectrometer with two ring detectors covering the scattering vectors q dynamical range of 0.005–0.55 Å^−1^ at 20.0 ± 0.50 °C. The intensity was determined in absolute units (cm^−1^) employing a vanadium standard. For the measurements, the solutions were put in Hellma quartz cells. For experiments with D_2_O and H_2_O as solvents, beam paths of 2 and 1 mm were used, respectively.

Primary treatment of the SANS data included corrections for the sample transmission, sample thickness and electronic noise by SAS program [46,47,48]. Incoherent (background) scattering was subtracted from the data by using blank solvents. Fitting of the scattering curves was performed by the program SasView (http://www.sasview.org/).

Scattering curves in the absence and at small n-decane concentration (21 mM) were fitted by a model of an elliptical cylinder. Two fitting parameters (equatorial and polar radii of cross-section) were used. Scattering curves at high-decane concentrations (90 and 210 mM) were fitted by a model of a charged core-shell ellipsoid (form-factor of a core-shell ellipsoid combined with Hayter–Penfold (Rescaled Mean Spherical Approximation, RMSA) structure factor). The fitting was performed in the following way: first, scattering curves at intermediate and high-scattering vectors (q > 0.05 Å^−1^) were fitted by a form-factor of a core-shell ellipsoid without structure factor. Four fitting parameters (equatorial and polar radii of core, thickness of shell at the equator and at the pole) were used. Then, the geometrical parameters of the microemulsion droplet were fixed, and fitting by a combination of form-factor and structure factor was employed. Scattering curves at intermediate n-decane concentrations (35 and 70 mM) were fitted by a mixture of elliptical cylinders and core-shell ellipsoids. No structure factor was used in this case. Four fitting parameters (equatorial and polar radii of cylinder cross-section, fraction of cylinders in the mixture, and equatorial radius of ellipsoid core) were used. The ratio of polar to equatorial radius and shell thickness of ellipsoid were fixed according to the data obtained from fits at higher n-decane concentrations.

### 2.5. Cryogenic Transmission Electron Microscopy (Cryo-TEM)

Cryo-TEM experiments were performed in the bright field mode at Titan Krios 60–300 TEM/STEM instrument (FEI) operated at 300 kV. A spherical aberration corrector (image corrector), a direct detection camera Falcon II (FEI), and post-column energy filter (Gatan) were used for image acquisition. An underfocus of the ob jective lens of 2–10 nm was used to achieve phase contrast. For acquisition of some images, Volta phase plates were additionally used to enhance the contrast. Micrographs were obtained in low dose mode with a total electron dose of less than 15 e/Å^2^. Digital Micrograph (Gatan) and TIA (FEI) software were used for the image processing. For preparation of the cryo-TEM samples the solution was deposited via the side port of the Vitrobot (FEI) onto the Lacey carbon-coated side of the 300-mesh copper TEM grid, blotted, plunged into liquid ethane and then transferred to the microscope for investigation.

## 3. Results and Discussion

In this article, we study the effect of hydrocarbon—n-decane—on mixed WLMs formed by a short-chain (C8) cationic surfactant C8TAB and a long-chain (C18) anionic surfactant potassium oleate. The concentrations of C8TAB and potassium oleate were fixed at 117 and 78 mM, respectively, which corresponds to the total surfactant concentration of 5.5 wt% and the molar ratio of C8TAB/potassium oleate equal to 1.5, meaning that cationic surfactant is in excess. Therefore, the mixed WLMs under study are positively charged.

### 3.1. Before the Addition of n-Decane

**Figure 1:**
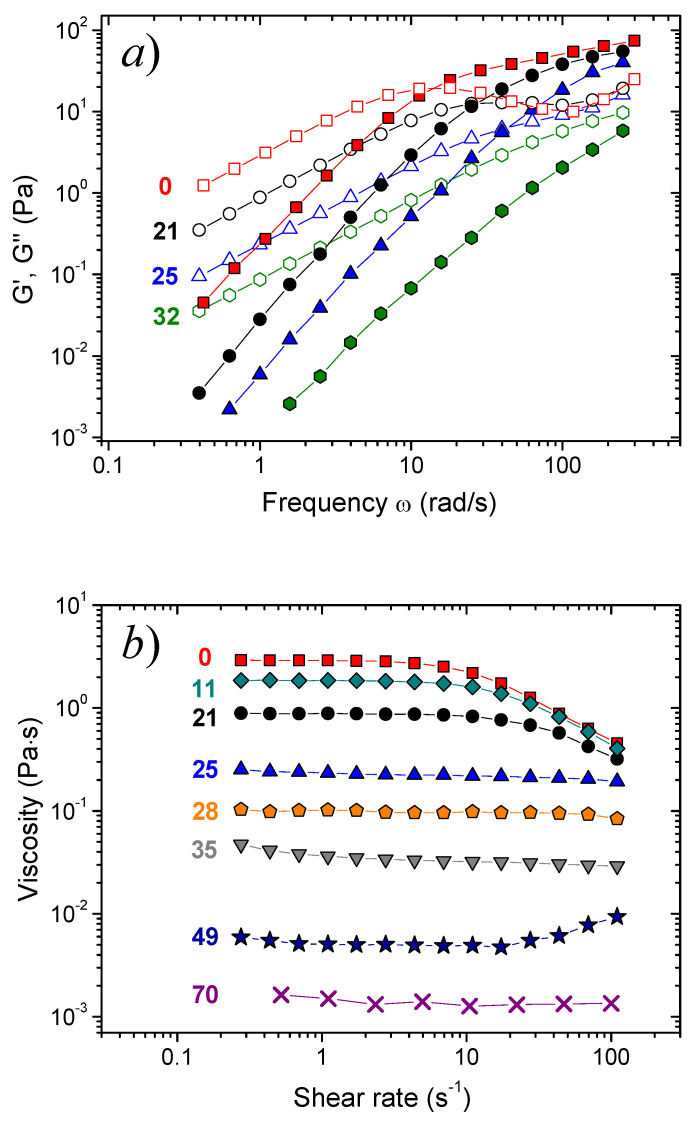
Frequency dependences of storage G’ (filled symbols) and loss G” (open symbols) moduli (**a**) and dependences of viscosity on shear rate (**b**) for aqueous solutions containing 117 mM C8TAB, 78 mM potassium oleate, and different amounts of added n-decane indicated in the figures (in mM), at 20 °C.

In the absence of hydrocarbon, a network of long entangled mixed C8TAB/potassium oleate WLMs is formed. This is confirmed by the frequency dependences of the storage (G’) and loss (G”) moduli (Figure 1a), which represent the viscoelastic behavior typical of wormlike micellar networks [49]: a high-frequency entanglement plateau is seen at the G’(ω) curve, and a cross-over point between the curves G’(ω) and G”(ω) is observed. The solution is characterized by rather high zero-shear viscosity (3 Pa·s) and shear-thinning behavior (Figure 1b) reminiscent of WLMs.

**Figure 2:**
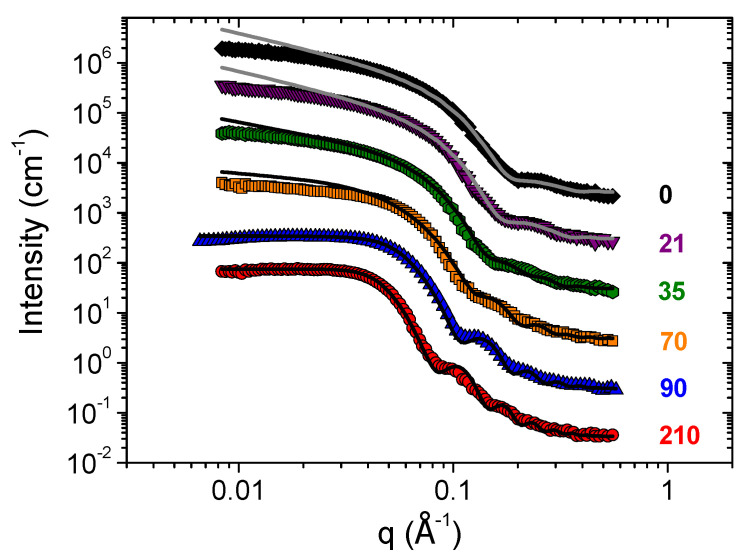
Scattering curves for 117 mM C8TAB and 78 mM potassium oleate solutions in D_2_O containing different amounts of added n-decane indicated in the figure (in mM), at 20 °C. Solid lines represent fits of the scattering curves by models of an elliptical cylinder (0 and 21 mM n-decane), a mixture of elliptical cylinders and core-shell ellipsoids (35 and 70 mM n-decane) and charged core-shell ellipsoid (90 and 210 mM n-decane). Parameters of fits are presented in Table 1.

Local cylindrical form of micelles is confirmed by SANS data (Figure 2). The scattering curve in the absence of n-decane is well-fitted by a form-factor of a rigid elliptical cylinder with radii R_1_ = 17 Å and R_2_ = 26.3 Å (Table 1). The minor radius R_1_ is comparable to the length of a fully extended potassium oleate alkyl tail, which is approximately 19 Å, and is 2 times larger than the length of C8TAB tail (9 Å). Similar situation was observed in negatively charged C8TAB/potassium oleate micelles at molar ratios of cationic to anionic surfactant lower than unity [12,25]. In the present paper, we demonstrate that even at the high excess of the short-chain cationic surfactant the cross-section radius of WLMs is controlled by the length of the longer surfactant tail. The elliptical cross-section of the micelles may be explained by the tendency of surfactant molecules to maintain optimal curvature in all parts of the surface of the micelles that leads to a non-uniform distribution and partial segregation of cationic and anionic surfactants within the cross-section. One can suggest that potassium oleate molecules are preferentially located at the minor ellipsoid axis, where the curvature is lower and is more favorable for them. At the same time, C8TAB molecules may be concentrated at the edges of the major axis, where the curvature is higher.

Micelles with elliptical cross-section were previously observed only in a few surfactant systems, including cationic/anionic surfactant mixtures (CTAB/sodium octyl sulfate [16], 1-dodecylpyridinium chloride (C12Pyr)/potassium oleate [50]) and cholesterol-based surfactants with added oils [37].

It should be noted that a deviation of the scattering curves from the rigid elliptical cylinder model is seen at low scattering vectors q < 0.025 Å^−1^ (Figure 2), which is most probably due to the intermicellar interactions, as well as to the micellar flexibility. These effects may be taken into account by the use of Pedersen–Schurtenberger model for micellar scattering [6], however, due to a limited range of low-q data and to the fact that C8TAB/potassium oleate micelles are charged and electrostatic interactions between them are not screened, it is hard to separate the effects of micellar flexibility and intermicellar interactions. At the same time, information about the elliptical cross-section of the micelles is contained in the high-q part of the scattering curves, which is not affected by the abovementioned factors, and is well-fitted by the rigid elliptical cylinder model.

C8TAB/potassium oleate WLMs under study are branched, since, according to the literature data, the concentration of C8TAB corresponds to the decreasing branch of viscosity curve [12,25]. A similar effect is usually observed for ionic surfactants in the presence of high concentrations of low-molecular weight salt, also corresponding to the regime where viscosity decreases upon addition of salt [51]. The presence of branching points is also confirmed by a very short characteristic relaxation time τ = 0.09 s (determined as τ = 1/ω_0_, where ω_0_ is a frequency at which G’ and G” intercept), since branching points can easily slide along the cylindrical body of the micelles allowing fast stress relaxation [52]. Branched micelles were previously observed in the mixtures of a similar cationic surfactant with longer C16 tail (cetyltrimethylammonium bromide, CTAB) and sodium oleate at the excess of the cationic surfactant [53].

Thus, before the addition of hydrocarbon the C8TAB/potassium oleate solution contains branched long WLMs that are entangled with each other.

### 3.2. After the Addition of n-Decane

The effect of different concentrations of hydrocarbon (C_h_) on the rheological properties and structure of C8TAB/potassium oleate micellar network is investigated. As seen from the viscosity curves (Figure 1b), the viscosity of the solutions continuously decreases upon addition of n-decane, and at 70 mM of hydrocarbon becomes equal to 0.0013 Pa·s, which is close to the viscosity of water. As a result, viscoelastic micellar network is transformed into a water-like liquid, and a total drop of viscosity exceeds 3 orders of magnitude. According to the literature data [24], this can be explained by breaking of WLMs due to solubilization of hydrocarbon in their micellar cores, and their transformation into microemulsion droplets. Though this effect is well-known for various wormlike surfactant micellar systems [22,23,24,25,26], there are almost no studies devoted to hydrocarbon-induced transitions of cationic WLMs or mixed cationic/anionic WLMs at the excess of a cationic surfactant. As shown above, the latter case corresponds to specific packing conditions for surfactant molecules, leading to the formation of worms with elliptical cross-section. Therefore, hydrocarbon-induced transitions and resultant microemulsion droplets are specific in such systems, as shown below.

**Figure 3:**
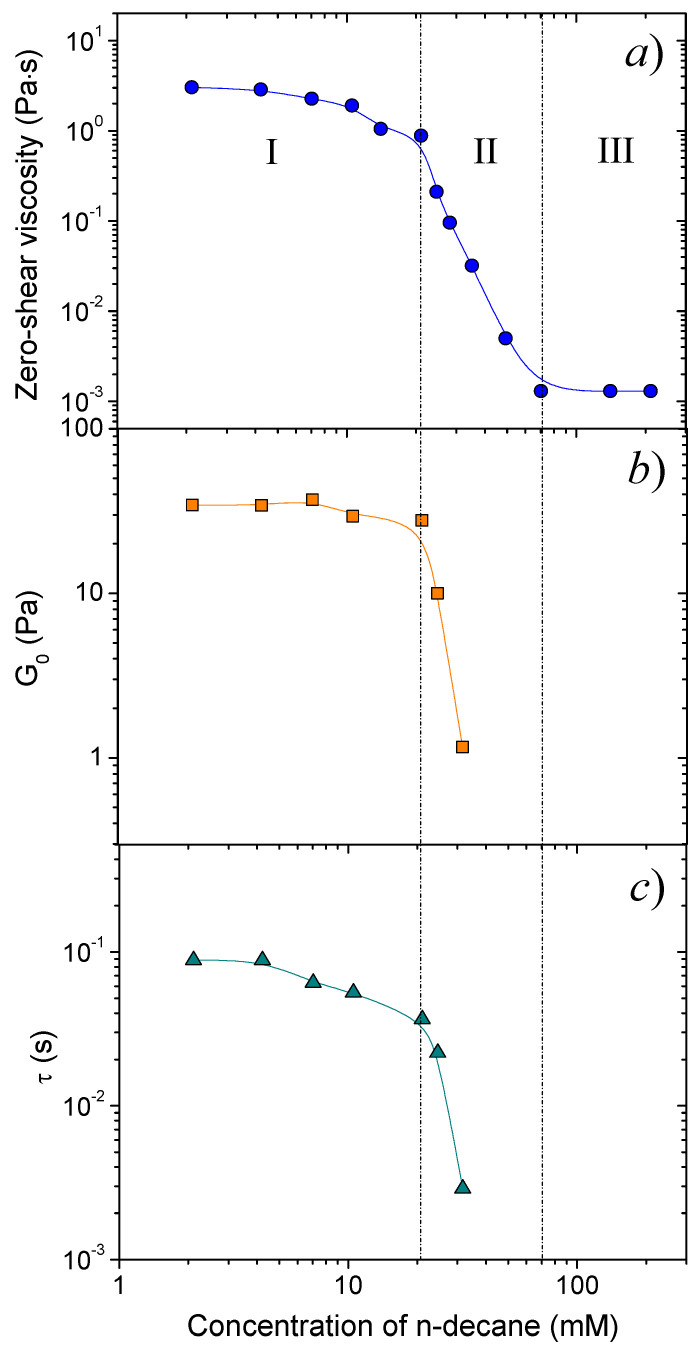
Dependences of zero-shear viscosity η_0_ (**a**), plateau storage modulus G_0_ (**b**), and terminal relaxation time τ (**c**) on n-decane concentration for 117 mM C8TAB and 78 mM potassium oleate aqueous solutions at 20 °C. In the case of absence of a well-defined plateau in the second range of hydrocarbon concentrations, the value of G’ at ω = 60 rad/s is used as G_0_.

Figure 3 presents the dependence of rheological properties of C8TAB/potassium oleate solutions on the concentration of added hydrocarbon C_h_. The dependence of zero-shear viscosity η_0_ on C_h_ (Figure 3a) can be divided into three regions, where the solutions have different properties: (I) “oil-swollen micellar network”, where the viscosity is rather high (η_0_ > 1 Pa·s), (II) “breakage of the micellar network”, where the viscosity drops drastically, (III) “ellipsoidal microemulsion”, where the viscosity is close to the value of water. Below we will consider all these three regimes in detail.

#### 3.2.1. Oil-Swollen Micellar Network

**Figure 4:**
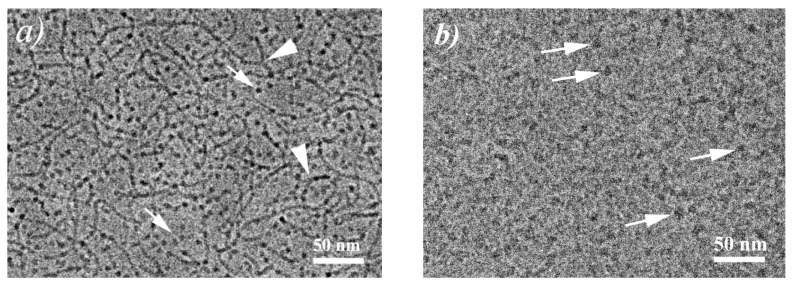
Cryo-TEM micrographs for aqueous solutions containing 117 mM C8TAB and 78 mM potassium oleate and 70 (**a**) or 90 (**b**) mM n-decane at 20 °C. Branching points of cylindrical micelles are indicated by arrowheads, microemulsion droplets—by arrows.

In the first region (at small hydrocarbon concentrations C_h_ ≤ 21 mM), the viscosity is 3 orders of magnitude higher than the viscosity of water (Figure 4a), and the solutions possess viscoelastic properties (Figure 1a) and shear-thinning behavior (Figure 1b). It indicates to the presence of an entangled micellar network. In this region, the dependences of the rheological parameters on n-decane concentration are characteristic of branched micelles [25]. First (at C_h_ < 4 mM), they stay constant, which is explained by preferential solubilization of hydrocarbon inside the branching points, which are the most unfavorable points within the micelle in terms of surfactant molecular packing. This increases the radius of the branching points, making them less energetically unfavorable, but does not change the scission energy and the length of the micelles, and, consequently, the rheological properties of the networks [25]. Then (at 4 mM < C_h_ < 21 mM), zero-shear viscosity (η_0_), plateau storage modulus (G_0_) and relaxation time (τ) start to decrease (Figure 3). This is due to solubilization of hydrocarbon in the other parts of the micelles, probably, mostly in the micellar end-caps [24], which leads to the shortening of the micelles and reduction of the rheological properties.

According to SANS data (Figure 2), in this regime, the oil-swollen cylindrical micelles retain the elliptical cross-section: at C_h_ = 21 mM the scattering curve is well-fitted by a model of an elliptical cylinder with the ratio of polar to equatorial radii close to the value found in the absence of hydrocarbon (Table 1).

Therefore, in the first region observed at small hydrocarbon concentrations an entangled network of oil-swollen WLMs with elliptical cross-section persists in the solutions. Addition of hydrocarbon induces a slight decrease of the rheological properties of the networks.

#### 3.2.2. Breakage of the Micellar Network

In the second region (at intermediate hydrocarbon concentrations: 21 mM < C_h_ ≤ 70 mM), the effect of n-decane drastically differs from that observed in the first region. The viscosity drops by 3 orders of magnitude down to the values close to the viscosity of water (Figure 3a). This is accompanied by a pronounced decrease of G_0_ and τ (Figure 3b,c) and further disappearance of viscoelastic properties and of shear-thinning (Figure 1). Therefore, the network of long interlaced micelles is broken due to solubilization of hydrocarbon in their cores.

In this region, the scattering curves are no longer fitted by a model of an elliptical cylinder, but are well approximated by a mixture of elliptical cylinders and ellipsoids (Figure 2). Upon increase of n-decane concentration, the fraction of ellipsoids in the mixture and their size (both equatorial and polar radii) increase, while the radii of elliptical cylinders do not change (Table 1). It indicates that cylindrical micelles are progressively broken into ellipsoidal microemulsion droplets upon addition of hydrocarbon. A similar effect has been recently observed by SANS in anionic potassium oleate WLMs [24], but in that case WLMs with circular cross-section were transformed into spherical microemulsion droplets.

Cryo-TEM micrograph of the solution corresponding to the end of this region (C_h_ = 70 mM) confirms the co-existence of cylindrical micelles and small microemulsion droplets (Figure 4a). For cylinders, multiple branching points are seen, indicating that WLMs remain branched in the course of the transition to microemulsion. From the cryo-TEM images, the size of microemulsion droplets may be estimated to be approximately 60 ± 10 Å, which qualitatively coincides with the SANS data (Table 1). However, this estimation is only qualitative due to the small droplets size and to the use of underfocus for image acquisition, which affects the visible size of the droplets.

It should be noted that this is the first direct confirmation by cryo-TEM that hydrocarbon-induced breaking of ionic wormlike micellar network proceeds via co-existence of cylindrical aggregates and microemulsion droplets. Previously, such a co-existence was observed by cryo-TEM only for a non-ionic surfactant pentaethylene glycol monododecyl ether (C12E5) in the presence of n-octane, but the transformation from WLMs to microemulsion droplets was detected upon decreasing both n-octane content and temperature [54].

Therefore, in the second region, mixed cationic/anionic WLMs continuously transform into elliptical microemulsion droplets, and upon increase of n-decane content, the fraction of droplets becomes higher, whereas the fraction of worms diminishes.

#### 3.2.3. Ellipsoidal Microemulsion

In the third region (at high hydrocarbon concentrations C_h_ > 70 mM), the viscosity of the solutions is nearly constant (0.0013 Pa·s) being close to the viscosity of water (Figure 3a). According to cryo-TEM data, all WLMs are disrupted, and only small microemulsion droplets are observed (Figure 4b). The size of the droplets is estimated to be approximately 80 ± 15 Å.

SANS curves in this range are well-fitted by a form-factor of a core-shell ellipsoid, combined with a structure factor which accounts for electrostatic repulsion between the droplets that are positively charged due to the excess of cationic surfactant content over the anionic one. Upon the increase of n-decane content, the size of microemulsion droplets increases due to solubilization of more hydrocarbon in their cores (Table 1). This coincides with the increase of the droplets size observed at the cryo-TEM images.

In contrast to larger emulsion droplets, which require more complicated preparation techniques and coalesce with time [55,56], the ellipsoidal microemulsion droplets obtained in this work form spontaneously and are thermodynamically stable [57], which is due to very low interfacial tension at the oil-water interface [58,59]. Ellipsoidal microemulsion droplets are usually observed for water-in-oil (W/O) microemulsions [60,61], but there are only several examples of oil in water (O/W) ellipsoidal microemulsion droplets discovered experimentally up to date [62,63]. Recently, ellipsoidal O/W droplets have been treated theoretically [64], and their existence in some systems has been predicted by molecular dynamics simulations [65].

**Figure 5:**
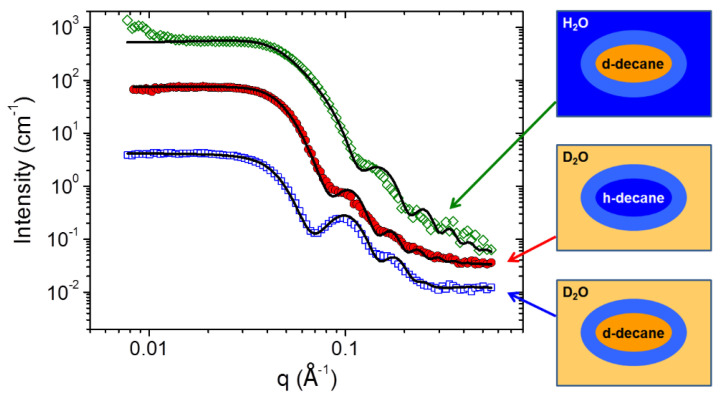
Scattering curves for 117 mM C8TAB and 78 mM potassium oleate solutions containing 210 mM of d-decane in H_2_O (diamonds), h-decane in D_2_O (circles) or d-decane in D_2_O (squares), at 20 °C. Solid lines represent fits of the scattering curves by a form factor of a charged core-shell ellipsoid (parameters of the fits are presented in Table 2).

In the present paper, the internal structure of ellipsoidal microemulsion droplets was for the first time investigated in detail by contrast variation SANS (Figure 5). For this, solutions were prepared with H_2_O or D_2_O as a solvent, and hydrogenated or deuterated n-decane as a hydrocarbon. The surfactants were always hydrogenated. In all cases, a model of a charged core-shell ellipsoid was implied, and scattering-length densities (SLDs) of the core, shell and surrounding solvent were varied accordingly. In the case of D_2_O and d-decane, the scattering curve is well approximated by scattering from an elliptical shell (Figure 5) with minor corrections due to the differences in SLDs of d-decane and D_2_O. For D_2_O and h-decane, the scattering is reminiscent of an ellipsoid, being the whole microemulsion droplet, with minor corrections due to its core-shell structure arising from different SLDs of h-decane and surfactants. For H_2_O and d-decane, the scattering is of a smaller ellipsoid, which represents the deuterated hydrocarbon droplet, slightly corrected by a core-shell structure due to different SLDs of surfactants and H_2_O.

**Figure 6:**
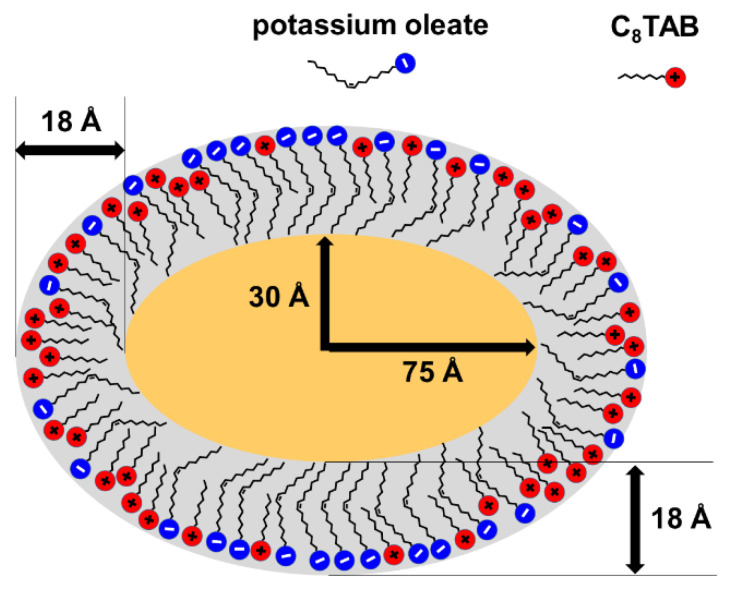
Schematic representation of an elliptical microemulsion droplet formed in aqueous solutions containing 117 mM C8TAB, 78 mM potassium oleate, and 210 mM n-decane.

For all three contrasts used, consistent geometrical parameters of the ellipsoids were obtained (Table 2), meaning that similar microemulsion droplets are formed when hydrogenated or deuterated compounds are used. The thickness of the shell is equal at the equator and at the pole (18–20 Å), and is close to the length of fully extended potassium oleate alkyl tail (19 Å). The core is a prolate ellipsoid with the ratio of polar to equatorial radii R_pol_/R_eq_ ≈ 2.5. These data suggest the following structure of the ellipsoidal microemulsion droplet (Figure 6): the core is an ellipsoidal droplet of hydrocarbon, which is surrounded by a mixed surfactant monolayer with constant thickness. In order for the non-spherical form of the droplet to be stabilized, a non-uniform distribution of cationic and anionic surfactants inside the layer should be realized. Since the thickness of the layer is uniform and is determined by longer oleate alkyl tails, one should expect that potassium oleate molecules are located both at the equator and at the poles. However, the curvature of surfactant layer is higher at the poles, which, from the point of view of surfactant packing, is more preferential for C8TAB molecules having shorter tail and bulkier polar head group. At the same time, the curvature is lower at the equator, which is favorable for oleate molecules characterized by longer tail and smaller head group. Therefore, C8TAB molecules may be preferentially concentrated at the poles stabilizing them, and more oleate molecules reside at the equator. From the volume of the core, the number of n-decane molecules solubilized in one microemulsion droplet is estimated to be at least 880, which is nearly 10-fold higher, than for n-dodecane/oleate microemulsion in the presence of KCl instead of C8TAB [24]. Thus, due to a rather large volume of the core, which is a result of the droplet being anisotropic and elongated in one direction, such microemulsions stabilized by mixed cationic/anionic surfactants have an advantage of higher payloads of hydrophobes.

Thus, in cationic/anionic surfactant mixtures, non-spherical forms of microemulsion droplets can be stabilized, because different surfactant species can easily re-distribute along the microemulsion surface and adapt to different curvatures at various points of non-spherical particle. This differs spontaneously formed microemulsions from larger emulsions, since the former are characterized by rather low interfacial tension of the surfactant monolayer, and, thus, smaller penalty for stabilizing the droplets of non-spherical shape, which have higher surface to volume ratio as compared to spherical droplets [66].

## 4. Conclusions

This paper demonstrates that in the mixture of two oppositely charged surfactants, strongly differing in the length of hydrophobic tails, a network of entangled wormlike micelles is formed at the excess of the short-chain cationic surfactant. As a result, the solutions possess unusual rheological behavior: high viscosity and pronounced viscoelastic properties combined with a very short relaxation time. Structural studies show that the cross-section of cylindrical micelles is not circular, but elliptical. Moreover, the shape of the microemulsion droplets that are formed as a result of the solubilization of a hydrocarbon in the micellar core is not spherical, but ellipsoidal. This unusual shape provides an optimum curvature of the surfactant monolayer both for long-chain surfactants (in the flatter part of the aggregate) and for short-chain surfactants (at the edges with higher curvature). At the same time, the opposite charge of the surfactants heads inhibits total segregation of both types of surfactants, which is evident from the constant thickness of the surfactant monolayer surrounding the hydrocarbon droplet.

The shape and size of the microemulsion droplets are of primary importance for many applications of surfactants in oil recovery. In dilute systems, most often the microemulsion droplets have a spherical shape because of surface tension, which dominates over all other forces at this size scale. Here, we demonstrate that in the mixture of two oppositely charged surfactants, strongly different in the length of hydrophobic tails, non-spherical (namely, ellipsoidal) thermodynamically stable microemulsion droplets are formed spontaneously. The stability of the droplets is important during the outflow of the broken fluid from the fracture, in order to prevent phase transformations which may occur under flow [67] and affect the clean-up process.

Moreover, usually, special methods like microfluidics, arrested coalescence, asymmetric polymer solidification, and evaporation-driven clustering [68] are used to get non-spherical emulsion droplets. Here, we demonstrate that simple mixing of surfactants strongly differing in the hydrophobic tail lengths provides an easy way to obtain spontaneously formed ellipsoidal droplets.

Such stable non-spherical droplets can be used a template for the synthesis of non-spherical nanoparticles, which may be further added to WLM solutions in order to increase their viscoelasticity [69,70]. Ellipsoidal nanoparticles have an advantage over spherical ones due to their high surface area, and, therefore, stronger interaction with WLMs. Therefore, the results of this study are quite promising for optimizing the application of mixed cationic/anionic WLMs in various fields, including oil recovery and template-assisted synthesis of non-spherical nanoparticles.

## Figures and Tables

**Table 1:** Parameters of aggregates formed in 117 mM C8TAB and 78 mM potassium oleate aqueous solutions in the presence of different n-decane concentrations obtained from SANS data.

N-Decane, mM	Elliptical Cylinder			Ellipsoidal Microemulsion Droplet			
	Fraction in the Mixture, %	Equatorial Radius R_eq_, Å	Polar Radius R_pol_, Å	Fraction in the Mixture, %	Equatorial Radius of Core R_eq_, Å	Polar Radius of Core R_pol_, Å	Thickness of Shell, Å
0	100	17	26.3	-	-	-	-
21	100	19	28.5	-	-	-	-
35	70	20	30	30	14	42	18
70	30	20	29	70	17	51	18
90	-	-	-	100	21	63	18
210	-	-	-	100	33	76	18

**Table 2:** Geometrical parameters of elliptical microemulsion droplets formed in 117 mM C8TAB, 78 mM potassium oleate, and 210 mM of n-decane aqueous solutions obtained from SANS data.

Solvent	Hydrocarbon	Equatorial Radius of Core R_eq_, Å	Polar Radius of Core R_pol_, Å	Thickness of Shell, Å
H_2_O	d-decane	35	77	18
D_2_O	h-decane	33	76	18
D_2_O	d-decane	29	73	20

## Abbreviations

EOR	enhanced oil recovery
WLM	wormlike micelle
C8TAB	n-octyltrimethylammonium bromide
SANS	small-angle neutron scattering
cryo-TEM	cryogenic transmission electron microscopy
